# A Personalized Navigation Route Recommendation Strategy Based on Differential Perceptron Tracking User's Driving Preference

**DOI:** 10.1155/2023/8978398

**Published:** 2023-01-04

**Authors:** Pengzhan Chen, Jihua Wu, Ning Li

**Affiliations:** Taizhou University, School of Intelligent Manufacture, Taizhou 318000, Zhejiang, China

## Abstract

With the increasing frequency of autonomous driving, more and more attention is paid to personalized path planning. However, the path selection preferences of users will change with internal or external factors. Therefore, this paper proposes a personalized path recommendation strategy that can track and study user's path preference. First, we collect the data of the system, establish the relationship with the user preference factor, and get the user's initial preference weight vector by dichotomizing the *K*-means algorithm. The system then determines whether user preferences change based on a set threshold, and when the user's preference changes, the current preference weight vector can be obtained by redefining the preference factor or calling difference perception. Finally, the road network is quantized separately according to the user preference weight vector, and the optimal path is obtained by using Tabu search algorithm. The simulation results of two scenarios show that the proposed strategy can meet the requirements of autopilot even when user preferences change.

## 1. Introduction

In recent years, with the rapid development of information and intelligent transportation systems, artificial intelligence (AI) has been widely used in transportation tools to provide a comfortable travel experience for drivers and passengers [1–3]. The vehicle Internet tends to share data, enabling vehicles to exchange learning experiences and improve decision-making capabilities. The individual models are trained on the basis of the data collected. By collecting these learning models from all vehicles, a comprehensive model can be further developed. This is of the great significance to the application of intelligent transportation system (ITS) in autonomous driving and traffic control.

At present, the existing path navigation and recommendation software in China, such as the Baidu map application and the Josiah Goddard map application, are all popular recommendation software, mainly based on the shortest time [4, 5] and the shortest distance [6–8], has been unable to meet the growing demand for personalized tourism.

Path recommendation systems usually recommend paths based on the optimization of distance or travel time cost functions. However, the shortest or fastest route is usually not chosen by the driver, so the driver may have different recommendations when traveling.

The personalized route recommendation strategy for each driver's route selection preference has attracted much attention. The personalized route recommendation strategy not only meets people's personalized needs but also solves the Blythe's paradox, which is widely existed in the current transportation network.

### 1.1. Literature Review

At present, the driver can get the current driving status of the vehicle through the OBD data. In reference [9], the authors proposed a monitoring system consisting of OBD and GPS, designated area, pricing scheme, and the relationship between other related policies. The OBD device is used to monitor engine operation and measure fuel consumption and emissions reliably. In reference [10], OBD is used to analyze the driving behavior data through the vehicle preloading equipment and the factors that affect the safe driving and establish the logistic expression model. In reference [11], OBD is used to monitor and prompt hydrocarbon (HC) emissions caused by a failure of the vehicle emission control system. In this paper, the OBD data are used to correlate with the driving user's path selection preferences. Then, the user's initial preference weight vector is obtained by the bisecting *K*-means algorithm clustering. In reference [12], Xiong et al. proposed a model predictive control optimal path optimization and tracking framework. First, the relationship between the vehicle and the reference road is established in the road coordinate system, and then, the safe lane under the high constraint environment is established by using the multilayer search method; path boundary and vehicle dynamics constraints are introduced to provide optimal control instructions.

In reference [13], the unsupervised classification method based on the bisecting *k*-means algorithm is applied to the data obtained in low-energy consumption proofread measurement of the online gas analyzer and microsensor. The good agreement with data from sensors validated the effectiveness of the proposed method. In [14], Zhao and othersproposed a reviewed block-based bag of words model using the bisection *K*-means clustering method which could significantly accelerate the process of codebook generation. In reference [15], a new algorithm based on graph mining is proposed and the bisecting *k*-means algorithm is used to find frequent terminology collection for the document. The Tabu search algorithm is a typical shortest path algorithm, which is used to calculate the shortest path from one node to other nodes and is widely used in path planning. In reference [16], the Tabu search algorithm is proposed to solve the problem of Web service composition. It adds a periodic diversification step, which is kept on the diversification step at the usual completion point. This approach combines path relinking with dynamic diversification strategies, providing more opportunities for future research. In [17], the Tabu search algorithm is used to improve the route planning strategy in urban areas under traffic congestion, which not only saves driving time but also reduces fuel consumption. In [18], the Tabu search algorithm is used to identify the current topology of the network and help identify the shortest path from the point of failure to the nearest operation source. The model improves efficiency consumption by 23% and bandwidth lifetime by 16%. How to make navigation more effective has been a research hotspot. In [19], Xie et al. proposes a new method of combining global path planning with local path planning, to provide an efficient solution for the unmanned surface vehicle (USV) path planning despite the changeable environment. The method solves the path planning problem with variable environments and is verified by simulations and experiments. In [20], Mei proposes an optimal tour guide path planning model based on an ant colony algorithm. The experimental results show that the proposed model and the optimal path planning algorithm are more optimized. In [21], rough set theory and a genetic algorithm is used in this study to solve the low efficacy and accuracy in robot path planning. Experimental results show that the proposed method can efficiently improve robot path planning. In [22], Chen et al. proposed a deep reinforcement learning algorithm for path planning, which has the comprehensive reward function of dynamic obstacle avoidance and goal approaching. The results show that the method can avoid moving obstacles in the environment, complete the planning task, and has a high success rate.

The personalized needs of users are not mentioned in these articles. In [23], Long et al. developed a novel route recommendation system to provide real-time personalized route recommendation for self-driving tourists, according to the specific preferences of users personalized access routes, not only to save the total tour time but also to meet their specific travel preferences. In [24], a dynamic route guidance method for driver's personalized needs is proposed. The user preference weight is given artificially, which has strong subjectivity and lacks objectivity and accuracy, but it provides a reference for the combination of personalized needs and path planning. In reference [25], a personalized path decision algorithm based on user preference is proposed, which lays a good theoretical foundation and framework for this study. But there are also limitations. The one-time sample trajectory data used in this paper needs a lot of complex mathematical calculations to obtain the data needed for clustering. The corresponding weight of user driving style obtained by fuzzy *c*-means clustering is fixed and one-sided, while in real life, the user driving preferences are not always the same and most of them are staged, so it cannot reflect the user's personalized driving preferences.

### 1.2. Contribution

As mentioned above, most traditional path planning schemes improve the speed and precision of path planning algorithmically, while ignoring the effect of user preferences [26–30] on path selection. In scenarios that combine user preferences and path planning, user preferences are unchanged by default. In real life, however, user preferences are not always the same. At different times, user preferences may change periodically due to various internal or external factors, such as age, physical condition, climate, and work. The type of preference may also change over time. At the same time, when the preference type is unchanged, the corresponding preference weights may also change.

Therefore, this paper proposes a personalized path planning strategy that can track and study user preferences. First, the preference type is associated with the OBD data and a threshold is used to select the user's current preference type. Then, the initial preference weight vector is obtained by the split-k-average algorithm. Finally, the differential perceptron is used to track and adjust the preference weights in real time, so that the optimal path can be recommended to meet the user's needs when the user's preferences change. In this paper, Nanchang is taken as the reference city of road network quantification, and the Tabu search algorithm is used to verify the effectiveness of the strategy.

In the light of the above, the contributions of the present document are summarized as follows:

1. To associate OBD with user preferences and get the initial preference weight vector by clustering
2. Considering the periodic change of preference, the change of two kinds of preference is summarized
3. Tracking the user preferences and fine-tuning the weights through the differential perceptron
4. Personalized quantization of road network, using the Tabu search algorithm to plan the optimal path
5. Taking Nanchang as a reference city, the simulation experiment is carried out to verify the effectiveness of the proposed strategy

## 2. System Implementation Review

**Figure 1:**
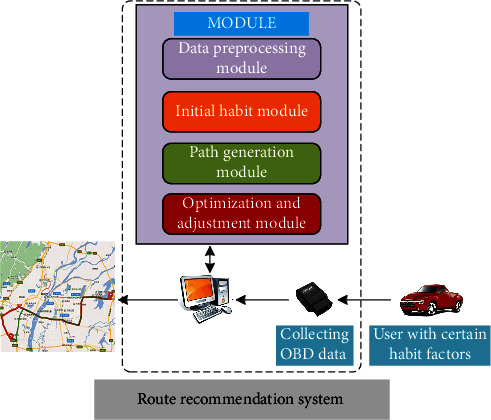
The illustration of path recommendation.

A personalized path recommendation system that can track and study the user's path selection preferences is proposed in this paper, as shown in Figure 1. The system mainly includes data preprocessing module, initial preference module, optimization and adjustment module, and path generation module. When the change of user preferences has been detected, the system will make use of the latest OBD data in the storage space to study independently offline until a path that can meet the user's current path selection preferences has been found.

**Figure 2:**
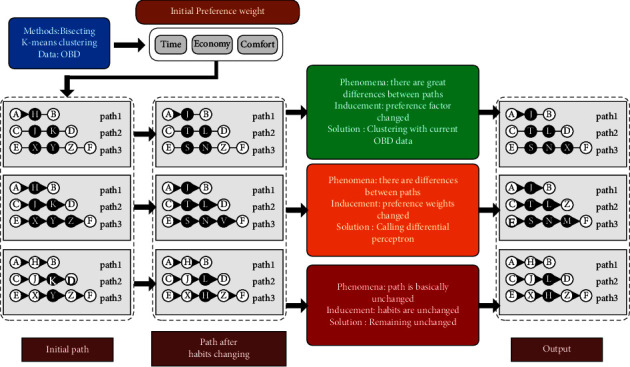
The illustration of recommendation under different circumstances.

A brief process of tracking and studying the path recommendation system is shown in Figure 2. In which, three preference factors of time, economy, and comfort are taken as the user's initial path selection preferences, and the path points marked in black are path offset points. The first case is that the preference factors change, the latest recorded OBD data are needed to redetermine them, and the initial driving preference weight is obtained by clustering. In the second case, the difference perceptron needs to be used for learning and correction because the preference factors remain unchanged and the corresponding weight values between the preference factors change. The third case is that in some road sections, the planned path is not consistent with the actual driving path. Since, the traffic network is real-time and dynamic, emergencies, such as traffic lights, pedestrians, and accidents, will affect the coincidence rate. Thus, in the actual situation, the planning path and the driving path are not exactly consistent. Therefore, within the allowable error range, in this case, it is considered that the user preference remains unchanged and it is considered as a sudden situation.

## 3. Path Recommendation System

### 3.1. Data Preprocessing Module

**Figure 3:**
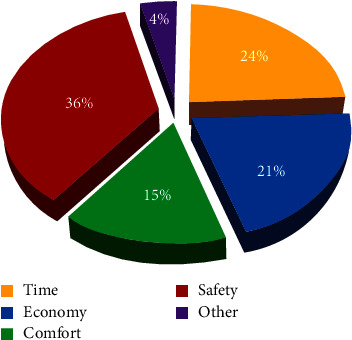
Results of habit factor sampling for surrounding car users.

Different self-driving users have different path selection preferences, so we conducted a questionnaire on the surrounding self-driving users and obtained the survey data shown in Figure 3. From Figure 3, we can see that four preference factors are affecting the route selection of self-driving users, namely, time, economy, comfort, and safety. Among them, the highest attention is paid to safety. In this paper, we choose these four preference factors as user preference sets.

To determine the type of user preference factors, OBD data and user preference are used to establish a connection in this paper [31]. The main function of OBD is to supervise the status of components related to emission control during the actual use of vehicles. In this paper, OBD status information, geographic location information, and trip record information are used to correlate user preference factors. The correlation process is as follows:

1. In the driving process, the greater the average speed of the user, the more the user attaches importance to time. In this paper, $\bar{v}$ is selected to be the relevance quantity (RQ) of a time-based user and $\bar{v}$ in any period time can be read directly from the server through the OBD terminal device.
2. The average fuel consumption (*AFC*) in the driving process is the most important indicator for economic users. The lower the value, the higher importance users attach to the economy. Therefore, *AFC* is chosen as the associated quantity of economic users in this paper. As there is no specific calculation method for *AFC* in the standard OBD II and EOBD protocol, it is necessary to use OBD related data for estimation.
3. The change rate of the relative position of the accelerator pedal (*CR*_*ap*_) is closely related to the comfortable user. The lower the (*CR*_*ap*_) value, the higher the user's attention to the comfort. Users who pay attention to comfort have good driving stability. They step on and loose slowly, and give oil smoothly. Therefore, *CR*_*ap*_ is chosen as the correlation quantity of comfortable users in this paper. Moreover, it can be obtained directly from the server by modifying the interval time parameters of the standard OBD II and EOBD protocol.
4. In the process of driving, excessive driving speed will bring a threat to the safety of users. Therefore, the maximum traveling speed of this period is taken as the correlation quantity of safe users in this paper. However, it can be read directly from the OBD terminal device.

The calculation process of each associated quantity is shown in Table 1.

Considering the laws and regulations related to safe driving and the opinions of professionals in the automobile industry, the threshold value of the average value of daily correlation volume is set, as shown in Table 2.

The maximum three items of *δ*_*i*_ are selected as the user's preference factors by the relevant degree *δ*_*i*_ of correlation and threshold calculation. As *k* = 3, the clustering effect is obvious, and to explain the change of preference factors conveniently, three preference factors are selected as the user's path selection preference each time. The relevant degree calculation formula is defined as follows:

(1) $$\begin{array}{c} \left\{\begin{array}{c} \delta_{i}=\frac{e^{\left|m_{i}-n_{i}\right|}}{e^{m_{i}}}\\ n_{i}=\frac{1}{N}\overset{N}{\underset{j=1}{\sum }}n_{ij} \end{array}\right.\left(n_{i}<2m_{i},i\in T,E,C,S\right)\text{.} \end{array}$$

### 3.2. Initial Habit Module

After the user preference factor is determined, the initial preference weight vector is obtained by clustering. Time, economy, and comfort are taken as examples and the bisecting *k*-means algorithm is used in clustering in this paper. To facilitate the clustering and effect display of OBD data, the data should be normalized before clustering. The partial results of some user OBD sampling data processed are shown in Table 3.

**Figure 4:**
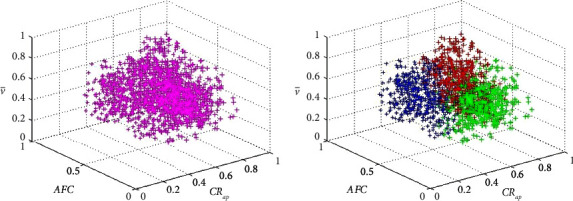
Bisecting *K*-means clustering results (above is before clustering and below is after clustering).

$\bar{v}$ , *AFC*, and *CR*_*ap*_ are used to establish the three-dimensional coordinate system, and then, the normalized data are divided into the bisecting *K*-means clustering, and the initial preference weight vector of users is obtained according to the clustering results. In order to make the clustering effect significant, here, select Δ*t*=1 min to be clustered, and the results are shown in Figure 4.

As can be seen from the figure, the value of red marked points $\bar{v}$-axis is generally higher than the other two categories. Due to the highest attention to time, the value $\bar{v}$ of time users is generally large. Therefore, the red marker class is a time-based feature point and The values of the *AFC*-axis of the points marked in green are generally smaller than those of the other two categories. Economic users pay the highest attention to fuel consumption, and the value of *AFC* is generally very small. Therefore, the green marker is the economic characteristic point. The value of *CR*_*ap*_-axis of blue marked points is generally smaller than the other two categories. Comfortable users pay the most attention to driving stability and the value of *CR*_*ap*_ is generally very small. Therefore, the blue marker is the comfort feature point. After the bisecting *K*-means clustering converges, the number of coordinate points belonging to three clustering centers is normalized to get the initial user preference weight vector *w*=(*w*_1_, *w*_2_, *w*_3_). The normalization formula is as follows:

(2) $$\begin{array}{c} w_{j}=\frac{n_{j}}{n_{1}+n_{2}+n_{3}}\left(j=1,2,3\right)\text{.} \end{array}$$

Among them, *n*_1_, *n*_2_, and*n*_3_ are the numbers of coordinate points belonging to the three clustering centers individually.

### 3.3. Path Generation Module

This part is mainly divided into two parts. The first part describes how to individualize and quantify the road network to establish a model for solving the optimal path. The second part shows how to calculate the optimal path by simulation.

#### 3.3.1. Road Network Quantification

To simplify the road model and calculation, referring to the regulations of highway technical engineering standard, urban road design code, urban road network planning index system, Nanchang urban traffic planning, and combining with the actual characteristics of the road network, Baidu map and the results of field investigation and verification, the roads in Nanchang are divided into the following five categories. To sum up, the road conditions in Nanchang are actual. The actual situation of each road is shown in Table 4.

In order to unify the quantitative standard and simplify the calculation of the optimal path with a genetic algorithm, the data in Table 3 are normalized and correlated. The process is as follows:

(3) $$\begin{array}{c} \left\{\begin{array}{c} \mathrm{Cos}t_{T}=\frac{1}{8}\left(0.6*\frac{300}{MS}+0.1*\frac{1}{RND}+0.3*\frac{4}{RS}\right)\text{,}\\ \mathrm{Cos}t_{E}=EC\text{,}\\ \mathrm{Cos}t_{C}=\frac{1}{8}*\frac{1}{RE}\text{,}\\ \mathrm{Cos}t_{S}=0.5*\frac{MS}{500}+0.5*RS\text{.} \end{array}\right. \end{array}$$

The data processed by formula (3) are shown in Table 5.

Based on the comprehensive consideration of different driving preferences and simplified calculation, the unit cost function is designed as follows:

(4) $$\begin{array}{c} U_{k}=f_{k}\left(c_{1},c_{2},\cdots ,c_{m}\right)=\overset{m}{\underset{i=1}{\sum }}\omega_{i}c_{i}\text{.} \end{array}$$

In this paper, each user has three main driving preferences, that is, the other three preference weights are set to zero, making *m*=3, so the cost of personalized quantification of the *K* segment road is as follows:

(5) $$\begin{array}{c} U_{Tk}=L_{k}\times f_{k}\left(c_{1},c_{2},c_{3}\right)=L_{k}\times \overset{3}{\underset{i=1}{\sum }}\omega_{i}c_{i}\text{.} \end{array}$$

#### 3.3.2. Tabu Search Algorithm

**Figure 5:**
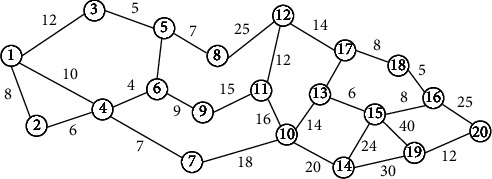
Road network model after personalized quantification.

After the recommendation system personalizes and quantifies the road network according to the user preference weight vector [32], the model becomes a classic problem of finding the optimal solution. In this paper, the Tabu search algorithm is used to calculate the optimal path according to the user's preference. Here, the model diagram shown in Figure 5 is selected to illustrate how the algorithm calculates the optimal path. In Figure 5, the cost consumption values of each point between 1 and 20 are calculated, respectively, by formula (5). After personalized quantification of the road network, finding the optimal path becomes a single source shortest path problem shown in Figure 5.

The process of using the Tabu search algorithm to get the optimal path of the model shown in Figure 5 is shown in Table 6. As can be seen from Table 6, the final optimal path is 1⟶4⟶7⟶10⟶13⟶15⟶16⟶20, and the total cost consumption is 88.

### 3.4. Optimization and Adjustment Module

In order not to affect the user's self-driving experience, it is stipulated that the system will conduct tracking and studying at night every day, and the preference tracking process is shown in Table 7. Due to the existence of emergencies, the consistency between the planned path and the actual driving path will not always be 100%. In the system, a threshold parameter is set for the coincidence degree. Before training every day, the system will filter the OBD data of the day and eliminate the data whose coincidence degree is higher than the threshold value. Then, the system recalculates the correlation degree *δ*_*i*_ of four correlation quantities according to the remaining OBD data of the day in the storage space and sorts them from small to large. If the correlation quantity corresponding to the minimum value of *δ*_*i*_ changes, the system determines that the preference factor changes, and clusters the latest data of the other three correlation quantities to get the initial weight vector again. On the contrary, the system judges that the preference factor has not changed, and calls the differential perceptron to fine-tune the weight vector. Because of the relatively small number of data samples on the same day, direct clustering by ignoring the previous data will enlarge the effect of change. Data clustering together will cover up smaller weight changes. Therefore, in this paper, we choose to call the differential perceptron to fine-tune to improve the accuracy.

The setting of the differential perceptron function is shown in Table 8. Taking OBD data and current weight vector as input, the maximum number of iterations is set as 2,000. The total length of five types of roads is calculated for the actual path and the planned path, respectively, and the total cost corresponding to the three preference factors is calculated for the two cases. Then, the change of the weight of each preference factor is calculated. Among which, *η* is the learning efficiency, take 0.003 to obtain the weight vector closest to the current user path selection preference through repeated iterative learning.

## 4. Experiment

Two types of preference change are proposed in this paper: one is the change of user preference factor and the other is the change of the weight of the same preference factor. To verify the effectiveness of the strategy proposed in this paper, Nanchang city is taken as a reference City, and simulation experiments are carried out in two scenarios, respectively. In scenario 1, the user preference factor changes. Assuming that the initial preference factor of user *A *is economy, comfort, and safety, then due to work reasons, time is more important than the economy, so the preference factor becomeseconomy, comfort, and safety. In scenario 2, the weight of the same user preference factor changes. Suppose that the initial preference factor of user *B* is time, economy, and comfort. Then, due to the financial crisis of the family, user *B* pays more attention to the economy and less attention to comfort. In these cases, 90% of the threshold value is used to determine whether it is an emergency or not.

### 4.1. Experiment 1

**Figure 6:**
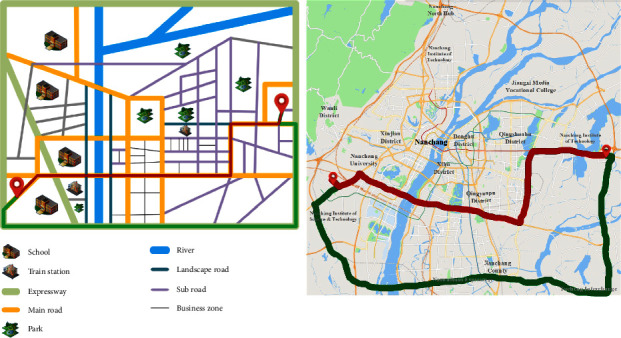
The initial planning path and the actual driving path before the adjustment in Experiment 1 (the left one is the model diagram and the right one is the actual map).

The preference factor of self-driving user *A* is economy, comfort, and safety, and the corresponding initial weight vector is obtained by the clustering algorithm, which is *w* = (0.48,0.14,0.38). After the preference factor of user *A* changes, the planned path and the actual driving path are shown in the red path and green path in Figure 6, respectively. It can be seen from the figure that due to the change of preference factors, the coincidence of the planning path and the actual path are very low, only 0.785%, indicating that the current planning strategy can no longer meet the personalized traveling needs of users.

After the personalized quantification of the road network *w*=(0.48,0.14,0.38), the specific generation values of economic consumption (TC), comfort consumption (EC), and safety consumption (CC) in two cases are shown in Table 9, respectively.

The total cost *C*_total_ of the actual path and the planned path can be obtained through classification and integration of the data of the road type in Table 9 and is shown in Table 10. As can be seen from Table 10, due to the change of preference type, the path types in the two cases are different, and the total cost is also very different, which is 12.737.

**Figure 7:**
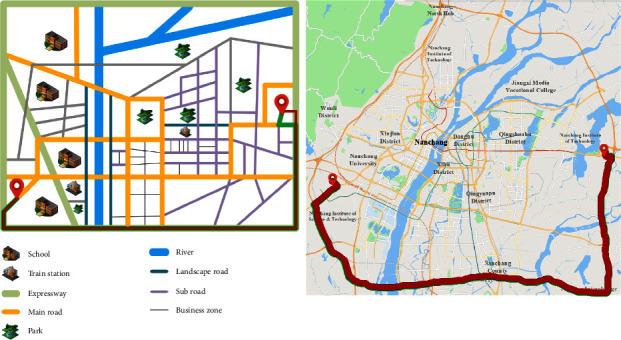
The adjusted initial planning path and actual driving path in Experiment 1 (the left diagram is the model diagram and the right one is the actual map).

When the system is tracking and studying at night, the correlation *δ*_*i*_ of four preference factors is calculated by formula (1). Among them, the correlation quantity of minimum *δ*_*i*_ changes from the previous time to economy. Therefore, the system determines that the user A's preference factor changes, and the current preference factor changes to time, comfort, and safety. The current weight vector is obtained by the bisecting *K*-means clustering. Finally, the planning path calculated by the Tabu search algorithm using personalized quantitative road network is shown in the red path in Figure 7.

As can be seen from Figure 7, after tracking and studying, the consistency between the planned path and the actual path is greatly improved, from 0.785% to 98%. Among them, the specific cost data in the two cases after adjustment are shown in Table 11.

The total cost *C*_*total*_ of the actual path and the planned path can be obtained through classification and integration of the data of the road type in Table 11 and is shown in Table 12 As can be seen from Table 12, the path types in the two cases are the same, and the total cost difference is only 0.081, which indicates that the system can still meet the user's path planning needs through tracking and adjusting after the user's preference factor changes.

### 4.2. Experiment 2

**Figure 8:**
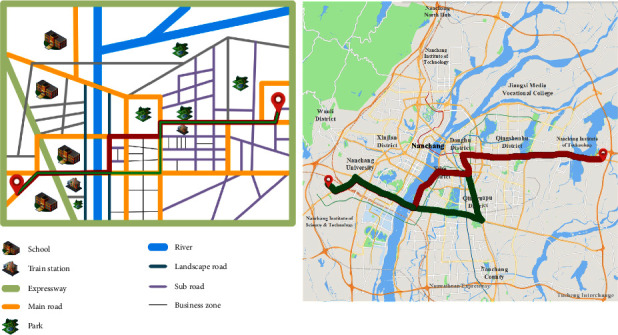
The initial planning path and the actual driving path before adjustment (the left one is the model diagram and the right one is the actual map).

The preference factor of self-driving user *B* is time, economy, and comfort, and the corresponding initial weight vector is *w*=(0.14,0.44,0.42). After user *B*'s preference weight changes, the planned path and the actual driving path are shown in red and green paths in Figure 8, respectively.

Due to the change of preference weight, the consistency between the planned path and the actual path is not high, which is 76.64%. It shows that the current planning strategy does not fully meet the user's personalized travel needs. To improve the degree of coincidence, we need to further improve the accuracy of the weight vector.

After the personalized quantification of the road network *w*=(0.14,0.44,0.42), the specific generation values of time consumption (TC), economic consumption (TC), and comfort consumption (EC) in two cases are shown in Table 13, respectively.

The total cost *C*_*total*_ of the actual path and the planned path can be obtained through classification and integration of the data of the road type in Table 13 and is shown in Table 14.

It can be seen from the table that, due to the change of preference weight, the path types in the two cases are the same, but the length is different, and the total cost difference is 2.149.

**Figure 9:**
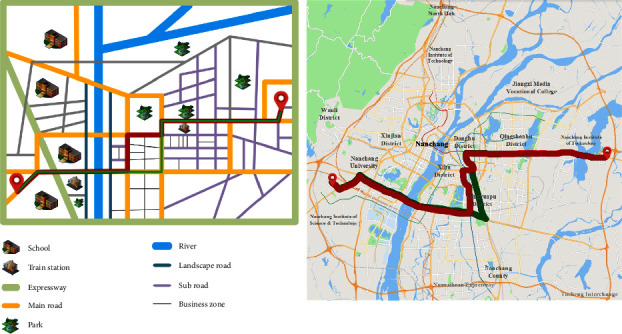
The initial planning path and the actual driving path before adjustment (the left one is the model diagram and the right one is the actual map).

Similarly, when the system is tracking and studying at night, the correlation *δ*_*i*_ of four preference factors is recalculated by formula (1). Among them, the correlation quantity of the corresponding minimum *δ*_*i*_ value does not change, which is safety. Therefore, the system will determine that the user B preference type has not changed but the weight changes, and call the differential perceptron to fine-tune, and finally, get the current weight vector *w*′=(0.15,0.51,0.34). Finally, the planning path calculated by the Tabu search algorithm using personalized quantitative road network *w*′ is shown in the red path in Figure 9.

As can be seen from Figure 9, after tracking and studying, the consistency between the planned path and the actual path has improved, from 76.64% to 84.58%. Among them, the specific cost data in the two cases after adjustment are shown in Table 15.

The total cost *C*_total_ of the actual path and the planned path can be obtained through classification and integration of the data of the road type in Table 15 and is shown in Table 16. As can be seen from Table 16, the path types in the two cases are the same, and the total cost difference is 1.199. Compared with studying before adjustment, the difference in total cost decreases and the coincidence increases. It shows that the system can also meet the user's path planning needs by tracking and adjusting when the user's preference weight changes.

## 5. Conclusion and Outlook

A personalized path recommendation strategy that can track and study the user's path selection preferences is proposed in this paper. In the case that the self-driving users do not know their preferences and their preferences will change periodically, the system first determines the main driving preference factors of users through the collected OBD data and obtains the initial preference weight vector through the bisecting *K*-means clustering. When the preference changes, the system judges according to the relevant parameters and adjusts the preference weight vector according to the situation. Then, the road network model is quantified according to the weight vector. Finally, the Tabu search algorithm is used to calculate the optimal path. In the two scenarios, through the comparison between the planned path and the actual path and the path before and after tracking and studying, the effectiveness of the strategy proposed in this paper is verified. The results show the consistency between the planned path and the actual path has improved from 76.64% to 84.58% after tracking and studying the user's personalized driving behavior. The adjusted total cost of initially planned route is 18.580, while actual path 19.779. The path types of the two cases are the same, and the total cost difference is only 0.081. It is proved that the strategy can also meet the personalized traveling needs of users when their preferences change.

To make the Tabu search algorithm not easily fall into local optimum when calculating the optimal path, the road network model in this paper is relatively simple. However, when the actual road network is larger and the route is more complex, the algorithm is easy to fall into local optimum. At the same time, only static factors rather than the real-time traffic situations in the road network are considered in this paper. In the future work, we should improve the accuracy of planning path algorithm or change the way of road network modeling, and add some dynamic factors to make the strategy proposed in this paper more universal.

## Figures and Tables

**Table 1:** The OBD data processing algorithm.

Algorithm 1: OBD data processing
**Input:** OBD data, *V*_*E*_=0.91, *M*_*M*_=28.27, *R*=8.314, *P*_*PG*_=6.17, *G*_*PP*_=4.536, *R*_*A*_=14.7
**Output:** $\bar{v},AFC,{CR}_{ap},V_{\max}$
1: **Begin**
2: **While** car running
3: Read and storage the values of $\bar{v}$, *CR*_*ap*_ and *V*_max_ every 6 seconds from OBD data
4: Read the values of, *L*, *R*_*PM*_, *M*_*AP*_, and *I*_*AT*_ every 6 seconds from OBD data
5: Calculate AFC
6: *M*_*AF*_=(*R*_*PM*_ × *M*_*AP*_/*I*_*AT*_/120) × (*V*_*E*_/100) × *E*_*D*_ × *M*_*M*_/*R*
7: *AFC*=(*R*_*A*_ × *P*_*G*_ × *G*_*PP*_ × *v*/3600 × *M*_*AF*_) × (1+*L*)
8: Storage *AFC*
9: **End while**
10: **End**

**Table 2:** Correlation threshold.

Relevant quantity	$\bar{v}$ (km/h)	*AFC* (L/100 km)	*CR*_*ap*_ (%)	*V*_max_ (km/h)
Threshold	40	8	10	60

**Table 3:** Partial OBD data after normalization.

Time	$\bar{v}$	*AFC*	*CR*_*ap*_	*V*_max_
08:08:34:25	0.1250	0.0090	0.0588	0.3600
08:08:40:25	0.0500	0.0068	0.5294	0.2010
08:08:46:25	0.2500	0.1109	0.4706	0.5212
08:08:52:25	0.3500	0.1267	0.8824	0.5842
08:08:58:25	0.3562	0.1154	0.5882	0.6124
08:08:64:25	0.6250	0.7467	0.6471	0.7653
08:09:00:25	0.8875	0.9887	0.4706	0.9102

**Table 4:** Performance of road networks at all levels.

Time	RND (km/km^2^)	RS (v/c)	RE (a.u.)	MS (km/h)	EC (RMB/km)
Expressway	0.42	0.42	0.40	100	0.74
Main road	1.31	0.68	0.35	60	0.51
Sub road	1.60	0.84	0.30	50	0.58
Landscape road	0.38	0.62	0.65	50	0.54
Business zone	1.75	1.15	0.22	40	0.62

**Table 5:** Road network costs at normalized levels.

Road classifications	*Cost*			
	Cos*t*_*T*_	Cos*t*_*E*_	Cos*t*_*C*_	Cos*t*_*S*_
*E*	0.31	0.74	0.31	0.32
*M*	0.40	0.51	0.36	0.49
*S*	0.47	0.58	0.42	0.58
*L*	0.36	0.54	0.19	0.58
*B*	0.62	0.62	0.57	0.74

**Table 6:** Tabu search algorithm solution process.

Routes	Total consumptions
1⟶1	0
1⟶2	8
1⟶3	12
1⟶4	10
1⟶3⟶5	17
1⟶4⟶6	14
1⟶4⟶7	17
1⟶3⟶5⟶8	24
1⟶4⟶6⟶9	23
1⟶4⟶7⟶10	35
1⟶4⟶6⟶9⟶11	38
1⟶3⟶5⟶8⟶12	49
1⟶4⟶7⟶10⟶13	49
1⟶4⟶7⟶10⟶14	55
1⟶4⟶7⟶10⟶13⟶15	55
1⟶4⟶7⟶10⟶13⟶15⟶16	63
1⟶4⟶7⟶10⟶13⟶17	60
1⟶4⟶7⟶10⟶13⟶17⟶18	68
1⟶4⟶7⟶10⟶14⟶19	85
1⟶4⟶7⟶10⟶13⟶15⟶16⟶20	88

**Table 7:** The habit tracking processing algorithm.

Algorithm 2: Habit tracking processing
**Input**: The latest OBD data, current habit weight vector *w*
**Output**: new habit weight vector *w*′=(*ω*_1_′,*ω*_2_′,*ω*_3_′)
1: **Begin**
2: Calculate *δ*_*i*_ with the latest OBD data, *i* ∈ *T*, *E*, *C*, *S*
3: **If** RQ of *δ*_*i*__min unchanged
4: *w*′= Improved differential perceptron (the last OBD data, *w*)
5: **Else**
6: Obtain *w*′ by reclustering the OBD data of the other three RQ
(7) **End if**
(8) **End**

**Table 8:** The improved differential perceptron algorithm.

Algorithm 3: Improved differential perceptron
**Input:** OBD data, habit weight vector
**Output:** Improved new habit weight vector *w*′=*w*_1_*w*_2_*w*_3_
11: **While ***n* ! = 2000 or ‖*w*′ − *w*‖ > *ε*
12: **For** each road classification do
13: Calculate the total length of the section in AP and PP respectively
14: $\left\{\begin{array}{c} L_{Ti}^{AP}=\underset{j=1}{\sum }L_{Ti}^{AP}\\ L_{Ti}^{PP}=\underset{j=1}{\sum }L_{j}^{AP} \end{array}\right.i\in T,E,C,S$
15: **End for**
16: **For** each habit factor **do**
17: Calculate the total cost in AP and PP respectively
18: $\left\{\begin{array}{c} U_{APi}=L_{Ti}^{AP}\underset{i=1}{\sum }\omega_{i}c_{i}\\ U_{PPi}=L_{Ti}^{PP}\underset{i=1}{\sum }\omega_{i}c_{i} \end{array}\right.i\in T,E,C,S$
19: Calculate weight change volume respectively
20: ∆*ω*=*η*(∑*U*_*APi*_ − ∑*U*_*PPi*_)*i* ∈ *T*, *E*, *C*, *S*
21: Calculate and normalize the new driving style weight
22: *ω*_*j*_^*i*^=*ω*_*j*_+Δ*ω* *j*=1,2,3
23: **End for**
24: *n* = *n* + 1
25: Re-quantify road network with *w*′
26: Replan the optimal path with the Tabu search algorithms
27: **End while**
28: Return *w*′=*w*_1_*w*_2_*w*_3_

**Table 9:** Adjustment of the first two paths in Experiment 1.

Paths	Road names	Types	ERL (km)	EC	CC	SC
Initially planned route	Wugong Mountain Avenue	*M*	3.4	0.832	0.171	0.633
	Xiangyun Avenue	*M*	7.6	1.861	0.383	1.415
	Changnan Avenue	*M*	13.4	3.280	0.675	2.495
	Changdong Avenue	*M*	9.4	2.301	0.474	1.750
	Ziyang Avenue	*M*	6.5	1.591	0.328	1.210
	Ziyang East Avenue	*M*	2.6	0.637	0.131	0.484
	Aviation City Avenue	*S*	0.51	0.142	0.030	0.112

Actual path	Wugong Mountain Avenue	*M*	3.3	0.808	0.166	0.615
	Circumferential Expressway	*E*	60.4	21.454	2.621	7.345
	Ziyang East Avenue	*M*	0.79	0.193	0.040	0.147
	Aviation City Avenue	*S*	0.51	0.142	0.030	0.112

**Table 10:** The total cost of the first two paths before adjusting in Experiment 1.

Paths	Types	*L* (km)	*U*_*Tk*_	*C*_total_
Initially planned route	*M*	42.90	20.652	20.936
	*S*	0.51	0.284	

Actual path	*M*	4.09	1.969	33.673
	*E*	60.4	31.420	
	*S*	0.51	0.284	

**Table 11:** Data of two paths after being adjusted in Experiment 1.

Paths	Road names	Type	ERL (km)	TC	CC	SC
Initially planned route	Wugong Mountain Avenue	*M*	3.3	0.937	0.166	0.243
	Circumferential Expressway	*E*	60.7	13.360	2.634	2.914
	Liu Cheng Street	*S*	0.82	0.274	0.048	0.071

Actual path	Wugong Mountain Avenue	*M*	3.3	0.937	0.166	0.243
	Circumferential Expressway	*E*	60.4	13.294	2.621	2.899
	Ziyang East Avenue	*M*	0.79	0.224	0.040	0.058
	Aviation City Avenue	*S*	0.51	0.170	0.030	0.044

**Table 12:** Total cost of two paths after adjustment in Experiment 1.

Paths	Types	L (km)	*U*_*Tk*_	*C*_*total*_
Initially planned route	*M*	3.3	1.346	20.647
	*E*	60.7	18.908	
	*S*	0.82	0.393	

Actual path	*M*	4.09	1.668	20.728
	*E*	60.4	18.815	
	*S*	0.51	0.245	

**Table 13:** The data of the first two paths before the adjustment in Experiment 2.

Paths	Road names	Types	ERL (km)	TC	EC	CC
Initially planned route	Wugong Mountain Avenue	*M*	3.4	0.190	0.763	0.514
	Xiangyun Avenue	*M*	8.1	0.454	1.818	1.225
	Riverside Avenue	*S*	5.6	0.368	1.429	0.988
	Hongcheng Road	*M*	3.7	0.207	0.830	0.559
	Jinggangshan Avenue	*M*	0.9	0.050	0.202	0.136
	Eight One Avenue	*M*	1.5	0.084	0.337	0.227
	Beijing West Road	*L*	2.3	0.116	0.546	0.184
	Beijing East Road	*L*	5.8	0.292	1.378	0.463
	Ziyang Avenue	*M*	6.5	0.364	1.459	0.983
	Ziyang East Avenue	*M*	1.5	0.084	0.337	0.227
	Aviation City Avenue	*S*	0.51	0.034	0.130	0.023

Actual path	Wugong Mountain Avenue	*M*	3.4	0.190	0.763	0.514
	Xiangyun Avenue	*M*	8.1	0.454	1.818	1.225
	Changnan Avenue	*M*	8.7	0.487	1.952	1.315
	Nanlian Road	*M*	2.4	0.134	0.539	0.363
	Jinggangshan Avenue	*M*	4.8	0.269	1.077	0.726
	Eight One Avenue	*M*	1.5	0.084	0.337	0.227
	Beijing West Road	*L*	2.3	0.116	0.546	0.184
	Beijing East Road	*L*	5.8	0.292	1.378	0.463
	Ziyang Avenue	*M*	6.5	0.364	1.459	0.983
	Ziyang East Avenue	*M*	1.5	0.084	0.337	0.227
	Aviation City Avenue	*S*	0.51	0.034	0.130	0.023

**Table 14:** The total cost of the first two paths before the adjustment in Experiment 2.

Paths	Types	*L* (km)	*U*_*Tk*_	*C*_*total*_
Initially planned route	*M*	25.6	11.328	17.418
	*L*	8.1	2.979	
	*S*	6.11	3.111	

Actual path	*M*	36.9	16.328	19.567
	*L*	8.1	2.979	
	*S*	0.51	0.260	

**Table 15:** Adjusted data of two paths in Experiment 2.

Paths	Road names	Types	ERL (km)	TC	EC	CC
Initially planned route	Wugong Mountain Avenue	*M*	3.4	0.204	0.884	0.416
	Xiangyun Avenue	*M*	8.1	0.486	2.107	0.991
	Changnan Avenue	*M*	6.4	0.384	1.665	0.783
	Yingbin North Avenue	*M*	3.9	0.234	1.014	0.477
	Fuhen Road	*M*	1.6	0.096	0.416	0.196
	Hongcheng Road	*M*	1.1	0.066	0.286	0.135
	Jinggangshan Avenue	*M*	0.9	0.054	0.234	0.110
	Eight One Avenue	*M*	1.5	0.090	0.390	0.184
	Beijing West Road	*L*	2.3	0.124	0.633	0.149
	Beijing East Road	*L*	5.8	0.313	1.597	0.375
	Ziyang Avenue	*M*	6.5	0.390	1.691	0.796
	Ziyang East Avenue	*M*	0.79	0.047	0.205	0.097
	Aviation City Avenue	*S*	0.51	0.036	0.151	0.073

Actual path	Wugong Mountain Avenue	*M*	3.4	0.020	0.884	0.416
	Xiangyun Avenue	*M*	8.1	0.049	2.107	0.991
	Changnan Avenue	*M*	8.7	0.052	2.263	1.065
	Nanlian Road	*M*	2.4	0.014	0.624	0.294
	Jinggangshan Avenue	*M*	4.8	0.029	1.248	0.588
	Eight One Avenue	*M*	1.5	0.009	0.390	0.184
	Beijing West Road	*L*	2.3	0.124	0.633	0.149
	Beijing East Road	*L*	5.8	0.313	1.597	0.375
	Ziyang Avenue	*M*	6.5	0.039	1.691	0.796
	Ziyang East Avenue	*M*	1.5	0.084	0.337	0.227
	Aviation City Avenue	*S*	0.51	0.036	0.151	0.073

**Table 16:** Adjusted total cost of two paths in Experiment 2.

Paths	Types	*L* (km)	*U*_*Tk*_	*C*_total_
Initially planned route	*M*	34.19	15.129	18.580
	*L*	8.1	3.191	
	*S*	0.51	0.260	

Actual path	*M*	36.9	16.328	19.779
	*L*	8.1	3.191	
	*S*	0.51	0.260	

## Data Availability

The data used to support the findings of this study are available from the corresponding author upon reasonable request (pzchen@tzc.edu.cn).
